# Retrospective comparative study of early versus delayed electroacupuncture intervention on neurological functional recovery in patients with traumatic spinal cord injury

**DOI:** 10.1097/MD.0000000000046511

**Published:** 2026-01-02

**Authors:** Rui Gong, Xinsheng Chen, Yuan Zhong, Xiaoling Huang, Ronghong Zhang, Tianhui Li, Zhongcheng Yang, Min Zhang

**Affiliations:** a Department of Acupuncture, Jiulongpo Hospital, Chongqing University of Chinese Medicine, Chongqing, China; b Laboratory of Gross Morphology, Experimental Teaching Management Center, Chongqing Medical University, Chongqing, China; c Department of Special Medical Service Centre, The First Affiliated Hospital of Chongqing Medical University, Chongqing, China.

**Keywords:** early intervention, functional independence, neurological recovery, Pain management, traumatic spinal cord injury

## Abstract

This study aimed to compare the effects of early versus delayed electroacupuncture (EA) intervention on neurological recovery in patients with traumatic spinal cord injury (TSCI), in order to clarify the optimal timing of EA and provide clinical evidence for targeted rehabilitation strategies. A retrospective cohort study was conducted including TSCI patients treated between March 2020 and April 2025. Based on the initiation time of EA, patients were divided into an early intervention group (EA within 2 weeks postinjury) and a delayed intervention group (EA > 2 weeks and ≤ 2 months postinjury). Baseline demographic and clinical characteristics, treatment parameters, and pre- and posttreatment outcomes were collected. Neurological function was evaluated using the American Spinal Injury Association (ASIA) motor and sensory scores and ASIA impairment scale (AIS) grade. Functional recovery was assessed by the functional independence measure (FIM) and modified Barthel Index (MBI), and pain was measured using the visual analogue scale (VAS). Statistical analyses were performed with SPSS 26.0, and *P* < .05 was considered significant. Ninety-two patients met the inclusion criteria (46 per group). Baseline characteristics were comparable between groups (*P* > .05). At 6 months, the early group showed a higher rate of AIS grade improvement (60.9% vs 37.0%, χ² = 5.126, *P* = .024) and a greater proportion achieving functional ambulation (AIS D–E: 65.2% vs 47.8%, χ² = 4.017, *P* = .045). Improvements in ASIA motor (19.3 ± 6.4 vs 14.2 ± 5.9, *P* = .001) and sensory scores (both *P* < .01) were significantly greater in the early group. Functional outcomes also favored early intervention, with higher gains in FIM (36.3 ± 8.1 vs 30.3 ± 7.9, *P* = .001) and MBI (37.2 ± 7.5 vs 32.2 ± 7.3, *P* = .001). Pain relief was more pronounced (VAS reduction: 3.6 ± 1.0 vs 2.7 ± 1.1, *P* < .001), and the overall complication rate was lower (21.7% vs 43.5%, *P* = .022). Early EA intervention significantly improves neurological and functional recovery, enhances pain relief, and reduces complications in TSCI patients. Compared with delayed treatment, early EA demonstrates superior efficacy and safety, supporting its early application in rehabilitation protocols.

## 1. Introduction

Traumatic spinal cord injury (TSCI) is a severe central nervous system disorder most commonly caused by falls from height, traffic accidents, and crush injuries. It often leads to partial or complete loss of sensory, motor, and autonomic functions, thereby profoundly impairing patients’ quality of life and hindering social reintegration.^[1]^ Epidemiological data indicate that the incidence of TSCI has been increasing annually, predominantly affecting young and middle-aged populations. Consequently, TSCI imposes a substantial socioeconomic burden on families and healthcare systems, posing a major public health challenge.^[2]^ Currently, conventional treatments for TSCI include emergency decompression surgery, spinal fixation, pharmacological therapy, and rehabilitation training. Nevertheless, neurological recovery remains limited, and the disability rate is persistently high.^[3,4]^ Promoting neurological recovery after TSCI has thus become a critical focus in the fields of neurotrauma and rehabilitation medicine.

With advances in rehabilitation, acupuncture has increasingly been integrated into multimodal therapeutic strategies for spinal cord injury. Electroacupuncture (EA), a technique that combines traditional acupuncture with modern electrical stimulation, offers advantages such as operational simplicity, reproducibility, and controllable stimulation intensity. A growing body of evidence suggests that EA promotes neurological repair and functional recovery through multiple mechanisms, including improving microcirculation in injured spinal cord regions, attenuating secondary inflammatory responses, inhibiting neuronal apoptosis, and enhancing neuroplasticity and axonal regeneration.^[5–7]^ Moreover, EA has shown beneficial effects on muscle tone regulation, nerve conduction, and functional performance in patients with TSCI, and is therefore widely applied in clinical practice.^[8,9]^ However, the optimal timing of EA intervention remains controversial. Some studies suggest that early intervention, when secondary injury processes are still evolving, may confer neuroprotective effects and facilitate better recovery. Others argue that delayed intervention, administered after the acute stress phase, may avoid interference with systemic responses while still yielding favorable outcomes during rehabilitation.^[10,11]^ The absence of high-quality clinical trials directly comparing intervention timings has limited the standardized application of EA in TSCI rehabilitation.

It is noteworthy that recovery from TSCI is a prolonged process, during which patients undergo distinct pathophysiological phases spanning the acute, subacute, and chronic stages. Whether EA should be initiated as early as possible after injury or reserved until the condition stabilizes remains unresolved. In practice, some rehabilitation centers favor early intervention to optimize the neural microenvironment and mitigate secondary injury, whereas others prefer delayed initiation due to safety and compliance concerns. The lack of direct comparative studies has left uncertainty regarding the relative efficacy of early versus delayed EA intervention.

Against this backdrop, the present retrospective study compared the effects of early and delayed EA intervention on neurological recovery in patients with TSCI. By systematically assessing neurological outcomes, functional independence, pain, and complications over a 6-month follow-up period, this study aimed to provide evidence-based insights into the impact of intervention timing, thereby contributing to the optimization of EA strategies in TSCI rehabilitation.

## 2. Materials and methods

### 2.1. Study population

This retrospective cohort study was approved by the Ethics Committee of the first affiliated Hospital of Chongqing Medical University (approval no. 2020-YS-045). We identified patients with TSCI admitted to the Department of Rehabilitation between March 1, 2020 and October 31, 2024, a window chosen to ensure completion of at least 6 months of follow-up; the final assessments concluded in April 2025. Of 128 patients screened, 92 met the eligibility criteria and were included in the analysis, then categorized by the timing of electroacupuncture (EA) initiation into an early intervention group (EA within 2 weeks postinjury; n = 46) and a delayed intervention group (EA > 2 weeks and ≤ 2 months postinjury; n = 46). The protocol received institutional approval, and, given the retrospective design, the requirement for informed consent was waived; all patient data were anonymized and managed with strict confidentiality to safeguard privacy.

#### 2.1.1. Inclusion criteria

Age between 18 and 65 years; diagnosis of TSCI in accordance with the American Spinal Injury Association (ASIA) criteria; patients or their legal guardians provided informed consent and signed the consent form; complete clinical data available, including demographic information, treatment protocol, and neurological function assessments.

#### 2.1.2. Exclusion criteria

Pathological spinal cord injury; concomitant severe craniocerebral injury, thoracoabdominal trauma, or other life-threatening injuries; pregnant or lactating women; severe cardiac, hepatic, renal, or hematological diseases; contraindications to electroacupuncture, such as the presence of a cardiac pacemaker; psychiatric disorders that hinder cooperation with treatment and assessment.

#### 2.1.3. Withdrawal and elimination criteria

Patients who discontinued treatment due to disease aggravation or severe adverse events during the intervention; patients lost to follow-up during the study period; incomplete clinical data that precluded accurate analysis.

### 2.2. Treatment protocol

All patients received standard rehabilitation therapy for TSCI, including: acute-phase surgical management: such as spinal decompression or internal fixation, with the surgical approach determined by the extent and type of injury; pharmacological treatment: including methylprednisolone sodium succinate (for eligible patients), dehydrating agents (e.g., mannitol), and neurotrophic agents (e.g., mecobalamin); rehabilitation training: comprising limb functional training (e.g., passive joint mobilization, active muscle strengthening), balance training, and activities of daily living (ADL) training. Sessions were conducted twice daily, 45 minutes each, 5 days per week, for a total of 3 months. On this basis, both groups received electroacupuncture (EA) at different time points.

#### 2.2.1. Early intervention group

Patients initiated EA treatment within 2 weeks postinjury. Acupoint selection followed these principles: Du Meridian Points: on the injured area, there are 1 to 2 points on each side of the spinous processes of the vertebrae (such as Dazhu, Mingmen, Weiyangguan, etc). Jiaji points: 1 to 2 bilateral Jiaji points located 0.5 cun lateral to the spinous processes adjacent to the injured level; acupoint selection along meridians: for upper limb paralysis, add Jianyu, Quchi, Waiguan, and Gongsun; for lower limb paralysis, add Jianguan, Zusanli, Yanglingquan, and Taiyang; for disorders of defecation and urination, add Shenshu, Xiaoliao, Qihai, and Guanyuan. Procedure: patients were placed in the supine or prone position depending on acupoint selection. After local skin disinfection, sterile disposable filiform needles (0.30 × 40 mm) were inserted. Following the elicitation of the “Deqi” sensation, electrodes of an electroacupuncture device (HuaTuo SDZ-II, Suzhou Medical Appliance Factory, China) were connected. A dilatational wave with a frequency of 2/100 Hz was applied, with current intensity adjusted to patient tolerance. Each session lasted 30 minutes, performed once daily, 5 times per week, for 3 consecutive months.

#### 2.2.2. Delayed intervention group

Patients initiated EA treatment between > 2 weeks and ≤ 2 months postinjury. The acupoint selection, procedure, frequency, and treatment duration were identical to those in the early intervention group.

### 2.3. Outcome measures and assessment methods

#### 2.3.1. Demographic and clinical characteristics

Baseline demographic and clinical data were collected, including age, sex, etiology of injury (e.g., traffic accidents, falls from height, heavy object trauma), injury level (cervical, thoracic, or lumbar), injury severity [classified according to the ASIA Impairment Scale as complete spinal cord injury (AIS grade A) or incomplete spinal cord injury (AIS grades B–D)], time from injury to hospital admission, and surgical time.

#### 2.3.2. Neurological and functional assessments

Neurological function was assessed using the ASIA scoring system, which includes motor score (maximum 100 points), light touch score (maximum 112 points), and pinprick score (maximum 112 points), along with AIS classification.

Functional independence was evaluated using the functional independence measure (FIM), covering self-care, sphincter control, transfers, locomotion, communication, and social cognition, with a total score of 126 points.

ADL were assessed using the modified Barthel Index (MBI), with a maximum score of 100 points, where higher scores indicate greater independence.

#### 2.3.3. Pain assessment

Pain intensity was evaluated using the visual analogue scale (VAS) at baseline and at 6 months posttreatment. Patients were asked to indicate their pain level on a 10-cm horizontal line, where 0 represented “no pain” and 10 represented “worst imaginable pain.” This method provides a simple and intuitive measure of pain severity and treatment response, facilitating timely adjustments to therapy and symptom relief.

#### 2.3.4. Complication monitoring

During treatment, complications such as urinary tract infection, pressure ulcers, DVT, and pulmonary infection were closely monitored and recorded. The incidence of complications was calculated, as these adverse events may impede recovery and worsen prognosis. Monitoring complication rates was therefore essential for evaluating both treatment efficacy and safety.

#### 2.3.5. Data collection and management

Data were primarily obtained from hospital medical records and outpatient follow-up documentation. Trained personnel used standardized case report forms to record demographic information (e.g., age, sex, etiology, injury level and timing), treatment details (e.g., initiation time of electroacupuncture, selected acupoints, stimulation parameters, treatment duration), and outcome measures (e.g., ASIA scores and AIS classification, FIM, MBI, VAS, and complication occurrence). Missing data were supplemented whenever possible through direct patient or caregiver contact or review of additional medical records. When data could not be retrieved, appropriate statistical techniques (e.g., case deletion, mean substitution, or multiple imputation) were applied depending on the type and extent of missingness.

For data management, all information was entered into an electronic Excel database with double-entry verification to ensure accuracy and completeness. Data cleaning and preprocessing were performed, including consistency checks and removal of erroneous entries. Standardization procedures were applied where necessary, such as normalization of ASIA and MBI scores, to facilitate subsequent statistical analyses. A dedicated data quality control team supervised the process regularly to maintain data reliability.

### 2.4. Statistical analysis

All statistical analyses were performed using SPSS version 26.0 (IBM Corp., Armonk). Continuous variables were tested for normality using the Kolmogorov–Smirnov test. Normally distributed data were expressed as mean ± standard deviation (‾x ± s) and compared using the independent-samples *t* test; nonnormally distributed data were expressed as median (P25, P75) and compared using the Mann–Whitney *U* test. Categorical variables were presented as frequencies and percentages and analyzed using the χ² test or Fisher exact test where appropriate. Within-group comparisons of ASIA motor and sensory scores, FIM, and MBI before and after intervention were performed using paired-samples *t* tests. The degree of AIS grade improvement between groups was compared using the χ² test, and relative risk (RR) with 95% confidence intervals (CI) was calculated. To control for potential confounders such as age, sex, injury level, and baseline AIS grade, a multivariate logistic regression analysis was conducted, with AIS grade improvement (≥1 level) as the dependent variable. All tests were 2-sided, and a *P*-value <.05 was considered statistically significant.

## 3. Results

### 3.1. Comparison of baseline characteristics

A total of 92 patients with TSCI who met the inclusion criteria were enrolled in this study. According to the timing of electroacupuncture intervention, patients were divided into an early intervention group (n = 46) and a delayed intervention group (n = 46). No significant differences were observed between the 2 groups in terms of demographic characteristics, injury features, or pretreatment neurological function and ADL scores (*P* > .05), indicating good baseline comparability. As shown in Table 1, the early intervention group consisted of 33 males (71.7%) and 13 females (28.3%) with a mean age of 41.8 ± 11.2 years, while the delayed intervention group included 31 males (67.4%) and 15 females (32.6%) with a mean age of 43.1 ± 10.7 years (sex: χ²=0.174, *P* = .676; age: *t* = 0.550, *P* = .584). There were no significant differences in injury level or ASIA grade between the 2 groups (cervical/thoracic/lumbar: 20/21/5 vs 22/20/4, χ²=0.293, *P* = .863; A/B/C/D: 8/10/20/8 vs 7/11/19/9, χ²=0.121, *P* = .990). The leading cause of injury was traffic accidents (56.5% in the early group vs 54.3% in the delayed group), followed by falls from height and other causes, with no significant difference between the 2 groups (χ²=0.182, *P* = .913). Pretreatment ASIA motor scores, light touch, pinprick sensation, FIM, and MBI scores did not differ significantly between the 2 groups (all *P* > .05). Detailed values are presented in Table 1.

**Table 1:** Baseline characteristics of patient.

Characteristic	Early intervention (n = 46)	Delayed intervention (n = 46)	Statistic	*P*-value
Demographics				
Age (yr, mean ± SD)	41.8 ± 11.2	43.1 ± 10.7	*t* = 0.550	.584
Sex (M/F)	33/13	31/15	χ^2^=0.174	.676
Injury characteristics				
Injury level (C/T/L)	20/21/5	22/20/4	χ^2^=0.293	.863
ASIA grade (A/B/C/D)	8/10/20/8	7/11/19/9	χ^2^=0.121	.990
Cause of injury [n (%)]				
Traffic accident	26 (56.5%)	25 (54.3%)	χ^2^=0.182	.913
Fall from height	14 (30.4%)	15 (32.6%)		
Other	6 (13.0%)	6 (13.0%)		
Baseline functional scores				
ASIA motor score	49.2 ± 7.1	48.5 ± 6.8	*t* = 0.490	.626
ASIA light touch	52.7 ± 5.9	52.0 ± 6.1	*t* = 0.497	.621
ASIA pinprick	54.1 ± 6.2	53.5 ± 5.9	*t* = 0.413	.681
FIM	52.6 ± 7.5	51.9 ± 8.0	*t* = 0.400	.690
MBI	35.9 ± 6.0	34.8 ± 6.2	*t* = 0.743	.460

ASIA = American Spinal Injury Association, C/T/L = cervical/thoracic/lumbar, FIM = functional independence measure; MBI = modified Barthel index.

### 3.2. Comparison of neurological function recovery

#### 3.2.1. ASIA impairment scale (AIS) improvement

At the 6-month follow-up, neurological function in both groups improved compared with baseline; however, the early intervention group demonstrated greater improvement. In the early intervention group, 28 patients (60.9%) showed an increase of ≥ 1 AIS grade from baseline, compared with 17 patients (37.0%) in the delayed intervention group, with the difference reaching statistical significance (χ²=5.126, *P* = .024). Specifically, in the early intervention group, 6 patients improved from AIS A/B to C, 12 from C to D, and 10 from D to E. In the delayed intervention group, 3, 8, and 6 patients demonstrated the same respective improvements. By the end of follow-up, the proportion of patients achieving functional ambulation (AIS D or E) was 65.2% in the early group, significantly higher than 47.8% in the delayed group (χ² = 4.017, *P* = .045). These findings indicate that early electroacupuncture intervention can significantly promote neurological recovery in patients with TSCI and increase the rate of AIS grade improvement. Detailed results are presented in Table 2.

**Table 2:** Comparison of AIS grade improvement between groups.

AIS grade	Early intervention (n = 46)	Delayed intervention (n = 46)	χ^2^/*t*	*P*-value
Baseline				
A	8 (17.4%)	7 (15.2%)	–	–
B	10 (21.7%)	11 (23.9%)	–	–
C	20 (43.5%)	19 (41.3%)	–	–
D	8 (17.4%)	9 (19.6%)	–	–
E	0 (0%)	0 (0%)	–	–
6 mo follow-up				
A	6 (13.0%)	6 (13.0%)	–	–
B	4 (8.7%)	6 (13.0%)	–	–
C	16 (34.8%)	21 (45.7%)	–	–
D	12 (26.1%)	9 (19.6%)	–	–
E	8 (17.4%)	4 (8.7%)	–	–
Improvement ≥ 1 grade [n (%)]	28 (60.9%)	17 (37.0%)	χ^2^=5.126	.024*
Proportion AIS D–E [n (%)]	20 (43.5%) → 30 (65.2%)	21 (45.7%) → 22 (47.8%)	χ^2^=4.017	.045*

AIS = ASIA impairment scale.

* *P* < .05 indicates statistical significance.

#### 3.2.2. ASIA score improvement

At the 6-month follow-up, both groups demonstrated significant improvements in ASIA motor and sensory scores compared with baseline; however, the early intervention group exhibited greater gains. For motor scores, the early intervention group increased from 49.2 ± 7.1 at baseline to 68.5 ± 8.3, with a mean improvement of 19.3 ± 6.4 points. In the delayed intervention group, scores increased from 48.5 ± 6.8 to 62.7 ± 7.9, with a mean improvement of 14.2 ± 5.9 points. The between-group difference was statistically significant (*t* = 3.586, *P* = .001). For sensory scores, in the early intervention group, light touch scores increased from 52.7 ± 5.9 to 68.2 ± 7.1, and pinprick scores increased from 54.1 ± 6.2 to 69.0 ± 7.4. In the delayed intervention group, light touch scores increased from 52.0 ± 6.1 to 63.8 ± 6.8, and pinprick scores from 53.5 ± 5.9 to 64.7 ± 7.0. Improvements in both light touch and pinprick scores were significantly greater in the early intervention group compared with the delayed group (light touch: *t* = 3.147, *P* = .002; pinprick: *t* = 3.335, *P* = .001). These findings indicate that early electroacupuncture intervention significantly promotes motor and sensory functional recovery in patients with TSCI, demonstrating superior efficacy over delayed intervention. Detailed data are presented in Table 3.

**Table 3:** Comparison of ASIA score improvement between the 2 groups.

Variable	Early intervention (n = 46)	Delayed intervention (n = 46)	*t* value	*P*-value
Motor score				
Baseline	49.2 ± 7.1	48.5 ± 6.8	0.490	.626
6 mo	68.5 ± 8.3	62.7 ± 7.9	3.586	.001*
Change from baseline	+19.3 ± 6.4	+14.2 ± 5.9	3.586	.001*
Light touch score				
Baseline	52.7 ± 5.9	52.0 ± 6.1	0.497	.621
6 mo	68.2 ± 7.1	63.8 ± 6.8	3.147	.002*
Change from baseline	+15.5 ± 5.8	+11.8 ± 5.6	3.147	.002*
Pinprick score				
Baseline	54.1 ± 6.2	53.5 ± 5.9	0.413	.681
6 mo	69.0 ± 7.4	64.7 ± 7.0	3.335	.001*
Change from baseline	+14.9 ± 6.0	+11.2 ± 5.7	3.335	.001*

* *P* < .05 indicates statistical significance.

### 3.3. Functional independence and activities of daily living comparison

Following treatment, both groups exhibited significant improvements in functional independence and ADL compared with baseline; however, the early intervention group demonstrated significantly greater gains than the delayed intervention group (*P* < .001), as summarized in Table 4. For the FIM, baseline scores were comparable between groups (early intervention: 52.1 ± 7.3 vs delayed intervention: 51.4 ± 7.6, *t* = 0.451, *P* = .653). At the 6-month follow-up, the early intervention group increased significantly to 88.4 ± 9.2, whereas the delayed intervention group reached 81.7 ± 8.7, with a statistically significant between-group difference (*t* = 3.626, *P* = .001). Compared with baseline, the early intervention group demonstrated a mean improvement of 36.3 ± 8.1 points, exceeding that of the delayed intervention group (30.3 ± 7.9 points; *t* = 3.626, *P* = .001). For the MBI, baseline scores were also similar (early intervention: 35.4 ± 6.0 vs delayed intervention: 34.6 ± 6.2, *t* = 0.609, *P* = .544). At 6 months, scores increased to 72.6 ± 8.1 in the early intervention group and 66.8 ± 7.7 in the delayed group, showing a significant difference (*t* = 3.619, *P* = .001). The improvement magnitude was greater in the early intervention group (37.2 ± 7.5 vs 32.2 ± 7.3; *t* = 3.619, *P* = .001). These results indicate that early electroacupuncture intervention substantially enhances functional independence and daily living capabilities in TSCI patients, outperforming delayed intervention.

**Table 4:** Comparison of functional independence and daily living ability between the 2 groups.

Variable	Timepoint	Early intervention (n = 46)	Delayed intervention (n = 46)	*t* value	*P*-value
FIM score	Baseline	52.1 ± 7.3	51.4 ± 7.6	0.451	.653
	6 mo	88.4 ± 9.2	81.7 ± 8.7	3.626	.001*
	Change from baseline	+36.3 ± 8.1	+30.3 ± 7.9	3.626	.001*
MBI score	Baseline	35.4 ± 6.0	34.6 ± 6.2	0.609	.544
	6 mo	72.6 ± 8.1	66.8 ± 7.7	3.619	.001*
	Change from baseline	+37.2 ± 7.5	+32.2 ± 7.3	3.619	.001*

FIM = functional independence measure, MBI = modified Barthel index.

* Indicates statistical significance.

### 3.4. Pain severity comparison

Regarding pain severity, baseline VAS scores were comparable between the 2 groups (early intervention: 5.9 ± 1.0 vs delayed intervention: 5.8 ± 1.1; *t* = 0.456, *P* = .649). At the 6-month follow-up, VAS scores decreased significantly in both groups, indicating that electroacupuncture effectively alleviates pain in TSCI patients.

Specifically, the early intervention group’s VAS score decreased to 2.3 ± 0.9, while the delayed intervention group decreased to 3.1 ± 1.0, with a significant between-group difference (*t* = 3.923, *P* < .001). The mean reduction in VAS was 3.6 ± 1.0 in the early group, significantly >2.7 ± 1.1 in the delayed group (*t* = 3.923, *P* < .001). Further analysis using a ≥ 3-point decrease in VAS to define “significant pain relief” showed that 36 patients (78.3%) in the early intervention group achieved significant relief, compared with 27 patients (58.7%) in the delayed group, demonstrating a statistically significant difference (χ²=4.186, *P* = .041). In summary, although baseline pain levels were similar, early electroacupuncture intervention provided more pronounced pain relief and a higher rate of clinically meaningful improvement compared with delayed intervention, as detailed in Table 5.

**Table 5:** Comparison of pain intensity between the 2 groups.

Outcome	Early intervention (n = 46)	Delayed intervention (n = 46)	Statistic	*P*-value
Baseline VAS	5.9 ± 1.0	5.8 ± 1.1	*t* = 0.456	.649
6-mo VAS	2.3 ± 0.9	3.1 ± 1.0	*t* = 3.923	<.001*
Change from baseline	−3.6 ± 1.0	−2.7 ± 1.1	*t* = 3.923	<.001*
Significant pain relief (VAS reduction ≥ 3), n (%)	36 (78.3%)	27 (58.7%)	χ^2^ = 4.186	.041*

VAS = visual analogue scale.

* Indicates statistical significance.

### 3.5. Comparison of complication incidence

During the follow-up period, both groups experienced a certain degree of perioperative and rehabilitation-related complications. In the early intervention group, a total of 10 patients (21.7%) developed complications, whereas 20 patients (43.5%) in the delayed intervention group experienced complications. The difference between the groups was statistically significant (χ²=5.217, *P* = .022), suggesting that early electroacupuncture intervention can significantly reduce the overall complication rate in TSCI patients. Specifically, the incidence of each type of complication was as follows: urinary tract infection occurred in 3 patients (6.5%) in the early group and 6 patients (13.0%) in the delayed group, with no statistically significant difference (χ²=1.091, *P* = .296); pressure ulcers occurred in 2 patients (4.3%) in the early group and 5 patients (10.9%) in the delayed group, also not statistically significant (χ²=1.486, *P* = .223); pulmonary infection occurred in 2 patients (4.3%) in the early group and 4 patients (8.7%) in the delayed group (χ²=0.906, *P* = .341); deep vein thrombosis (DVT) occurred in 1 patient (2.2%) in the early group and 3 patients (6.5%) in the delayed group (χ²=0.957, *P* = .328); other mild symptoms (dizziness, nausea, etc) occurred in 2 patients (4.3%) in both groups (χ²=0.000, *P* = 1.000). These results, as shown in Table 6, indicate that the overall complication rate was significantly lower in the early intervention group compared to the delayed group. Although the incidence of individual complication types did not reach statistical significance, the overall trend suggests that early electroacupuncture intervention may have a beneficial effect on patient safety.

**Table 6:** Comparison of complication incidence between the 2 groups.

Complication type	Early intervention (n = 46)	Delayed intervention (n = 46)	Statistic	*P*-value
Urinary tract infection	3 (6.5%)	6 (13.0%)	χ^2^ = 1.091	.296
Pressure ulcer	2 (4.3%)	5 (10.9%)	χ^2^ = 1.486	.223
Pulmonary infection	2 (4.3%)	4 (8.7%)	χ^2^ = 0.906	.341
DVT	1 (2.2%)	3 (6.5%)	χ^2^ = 0.957	.328
Other mild symptoms	2 (4.3%)	2 (4.3%)	χ^2^ = 0.000	1.000
Total complication rate	10 (21.7%)	20 (43.5%)	χ^2^ = 5.217	.022*

DVT = deep vein thrombosis.

* *P* < .05 indicates statistical significance.

## 4. Discussion

This study retrospectively compared the effects of early versus delayed electroacupuncture (EA) intervention on the rehabilitation of patients with TSCI. The results demonstrated that both groups exhibited improvements in neurological function and ADL after 6 months of follow-up; however, the magnitude of improvement was significantly greater in the early intervention group. Specifically, the proportion of patients with an ASIA impairment scale (AIS) improvement of ≥ 1 grade was significantly higher in the early intervention group, and the percentage of patients achieving functional ambulation (AIS D or E) was also markedly higher. Moreover, the early intervention group showed greater improvements in ASIA motor and sensory scores, FIM, and MBI scores. In addition, patients in the early intervention group experienced better postoperative pain relief, with a higher proportion achieving a ≥ 3-point reduction in VAS scores, and a lower overall incidence of complications. These findings suggest that early EA intervention may provide superior benefits in promoting neurological recovery, enhancing quality of life, and reducing adverse events.

First, our findings support the notion that early intervention facilitates neurological recovery. The early phase after spinal cord injury is characterized by secondary injury processes, including inflammation, oxidative stress, and apoptosis, which can further exacerbate neural damage and functional deficits.^[12]^ Preclinical studies have demonstrated that electroacupuncture (EA) exerts neuroprotective effects by modulating neurotrophic factors, inhibiting inflammatory cytokine release, and improving local microcirculation, thereby creating a favorable microenvironment for neural repair.^[13–15]^ Moreover, experimental evidence suggests that early EA can enhance synaptic plasticity, promote axonal regeneration, and reduce neuronal apoptosis, resulting in improved structural and functional recovery.^[16,17]^ These findings indicate that initiating EA early after injury may help to attenuate secondary injury cascades, protect residual neurons, and ultimately enhance long-term neurological outcomes.

Second, the observed advantages of early intervention in ADL (FIM and MBI scores) and pain relief suggest that EA may not only support neurological recovery but also alleviate chronic neuropathic pain by improving sensory conduction and promoting neural plasticity. Previous studies have demonstrated that EA can activate central analgesic pathways and modulate the excitability of dorsal horn neurons, thereby reducing pain.^[18,19]^ Pain relief may further encourage patient participation in rehabilitation, enhancing functional recovery and establishing a positive feedback loop.

Notably, the incidence of complications in the early intervention group was significantly lower than that in the delayed group. This may be related to early functional recovery and improved mobility, which reduce the risk of pressure ulcers, urinary tract infections, and lower limb DVT. The effects of EA on autonomic regulation, bladder function, and circulation may also indirectly contribute to the lower complication rate.^[20,21]^

Despite the clinical relevance of these findings, several limitations should be acknowledged. First, this study was a single-center retrospective analysis with a limited sample size, which may introduce selection bias due to the nonrandomized nature of patient inclusion and the reliance on existing medical records. Second, as with all retrospective studies, there is a risk of information bias, particularly related to incomplete or inconsistent documentation of clinical variables and follow-up data. Third, although we attempted to control for major confounding factors such as age, sex, injury level, and baseline AIS grade through multivariate analysis, residual confounding from unmeasured or inadequately recorded variables (e.g., rehabilitation adherence, nutritional status, or comorbidities) cannot be completely excluded. Fourth, the timing of electroacupuncture intervention was only categorized into 2 broad groups – “early (within 2 weeks)” and “delayed (>2 weeks and ≤ 2 months)” – without further stratification to define the precise optimal intervention window. Fifth, the follow-up duration was limited to 6 months, preventing evaluation of long-term functional recovery and the durability of treatment effects. Finally, the underlying mechanisms of electroacupuncture require further elucidation through multicenter prospective randomized controlled trials and mechanistic studies incorporating neuroimaging and molecular biology.

In conclusion, this study demonstrates that early EA intervention is more effective than delayed intervention in promoting neurological recovery, improving ADL, relieving pain, and reducing complications in patients with TSCI. Future large-scale, multicenter, prospective studies are warranted to validate these findings and to investigate the optimal timing and underlying mechanisms of EA, thereby providing stronger evidence for TSCI rehabilitation.

## Author contributions

**Conceptualization:** Rui Gong, Xinsheng Chen, Yuan Zhong, Xiaoling Huang, Ronghong Zhang, Tianhui Li, Min Zhang.

**Data curation:** Rui Gong, Yuan Zhong, Xiaoling Huang, Tianhui Li, Min Zhang.

**Formal analysis:** Rui Gong, Xinsheng Chen, Yuan Zhong, Xiaoling Huang, Ronghong Zhang, Tianhui Li, Zhongcheng Yang, Min Zhang.

**Funding acquisition:** Xinsheng Chen, Yuan Zhong, Min Zhang.

**Investigation:** Xiaoling Huang, Ronghong Zhang, Min Zhang.

**Writing – original draft:** Xinsheng Chen, Zhongcheng Yang, Min Zhang.

**Writing – review & editing:** Xinsheng Chen, Zhongcheng Yang, Min Zhang.
